# Essential Role of Interferon Response in Containing Human Pathogenic Bourbon Virus

**DOI:** 10.3201/eid2507.181062

**Published:** 2019-07

**Authors:** Jonas Fuchs, Tobias Straub, Maximilian Seidl, Georg Kochs

**Affiliations:** University of Freiburg Medical Center, Faculty of Medicine, University of Freiburg, Freiburg, Germany

**Keywords:** Bourbon virus, Thogotoviruses, Orthomyxoviruses, tickborne diseases, arboviruses, zoonoses, innate immunity, interferon, antiviral treatment, viruses, vector-borne diseases

## Abstract

Bourbon virus (BRBV) is a recently discovered tick-transmitted viral pathogen that is prevalent in the Midwest and southern United States. Since 2014, zoonotic BRBV infections have been verified in several human cases of severe febrile illness, occasionally with fatal outcomes, indicating a possible public health threat. We analyzed the pathology of BRBV infection in mice and found a high sensitivity of the virus to the host interferon system. Infected standard laboratory mice did not show clinical signs or virus replication. However, in mice carrying defects in the type I and type II interferon system, the virus grew to high titers and caused severe pathology. In cell culture, BRBV was blocked by antiviral agents like ribavirin and favipiravir (T705). Our data suggest that persons having severe BRBV infection might have a deficiency in their innate immunity and could benefit from an already approved antiviral treatment.

In the past 4 years, several reports from the Midwest and southern United States have described the detection of a new viral pathogen, called Bourbon virus (BRBV), associated with severe febrile illness (*1*–*4*). The isolation of BRBV from patients with a history of tick bites was unexpected, because BRBV belongs to tick-transmitted viruses of the genus Thogotoviruses, which are largely unknown in the United States. However, recent tick surveillance campaigns confirmed the prevalence of BRBV in the affected region (*5*,*6*).

Thogotoviruses are influenza virus–like arboviruses with a segmented RNA genome; they are frequently isolated in Africa, the Middle East, Asia, and southern Europe and are usually associated with diseases in livestock (*7*). BRBV is genetically most similar to Dhori virus (DHOV) from India (*8*). Although serologic surveys suggest the occurrence of zoonotic transmission of Thogotoviruses, few human cases have been well-documented (*9*,*10*). However, in laboratory mice, Thogotoviruses show an aggressive systemic infection affecting mainly the liver, lungs, and spleen, leading to a fatal acute hepatitis. This severe disease progression is accompanied by a massive induction of interferon (IFN) α without an apparent protective effect (*11*,*12*).

We conducted our study with the aim to evaluate the virulence and pathogenesis of BRBV in vivo. Furthermore, we assessed the antiviral effect of the host IFN system on BRBV replication.

## Materials and Methods

### Biosafety and Animal Ethics

Because of the unknown health risk associated with the human BRBV isolate, all work with infectious virus was performed under Biosafety Level 3 conditions. The animals were handled in accordance with guidelines of the Federation for Laboratory Animal Science Association and the national animal welfare body of Germany. Animal experiments were performed in compliance with animal protection laws in Germany and approved by the local animal welfare committee (Regierungspraesidium Freiburg, permit no. G-15/127).

### Cells and Viruses

We cultivated human lung epithelial A549 cells (ATCC CCL-185), human hepatoma Huh7 cells (*13*), human cervix carcinoma HeLa cells (ATCC CCL-2), African green monkey kidney Vero cells (ATCC CCL-81), and transformed human dermal fibroblast cultures (*14*) in Dulbecco modified eagle medium supplemented with 10% fetal calf serum at 37°C and 5% CO_2_. We treated Huh7 cells with recombinant human IFN-α2a (PBL Assay Science, https://www.pblassaysci.com) or recombinant human IFN-γ (R&D Systems, https://www.rndsystems.com) 16 h before and 2 h after infection. To test antivirals, we treated Huh7 cells with ribavirin (Sigma-Aldrich, https://www.sigmaaldrich.com) or favipiravir/T-705 (BioVision, https://www.biovision.com) 2 h after infection. To evaluate the cytotoxicity of these substances, we treated uninfected cells for 48 h with the maximum doses as described. Afterward, we assessed the viability of the cells by measuring the activity of lactate dehydrogenase in the cell culture supernatant (Pierce LDH Cytotoxicity Assay Kit; ThermoFisher, https://www.thermofisher.com). To establish a positive control, we treated cells with the lysis buffer provided by the manufacturer.

### Viruses and Infection

BRBV (strain NR-50132/ATCC VR-1842) (*1*) was kindly provided by Amy J. Lambert of the Centers for Disease Control and Prevention (Fort Collins, CO, USA). We produced virus stocks of BRBV, DHOV (strain India/1313/61) (*15*), and Thogoto virus (THOV) (strain SiAr126) (*16*) on Vero cells. To perform growth kinetics, we infected the cells with BRBV at a multiplicity of infection (MOI) of 0.001 in Dulbecco modified eagle medium with 1% fetal calf serum and 20 mmol/LM HEPES. We harvested supernatants at the indicated time points. We stored virus-containing supernatants at −80°C, and we determined viral titers by using plaque assay on Vero cells.

### Animal Infections

We purchased wild type C57BL/6 mice from Janvier Labs (https://www.janvier-labs.com). We bred mice with defects in the IFN pathway (*17*) in house. We conducted all experiments by using sex- and age-matched animals (7–9 week-old mice). We infected animals intraperitoneally with BRBV or DHOV diluted in 100 µL PBS with 0.3% bovine serum albumin. Depending on the experimental setup, we measured weight, survival, and clinical score daily for each animal (Appendix). We euthanized the animals by using cervical dislocation at the indicated time points. To determine survival of the animals after infection, we euthanized animals if the weight loss was >25% or the mice showed severe clinical signs. We harvested organs (liver, lung, spleen, and kidney) at day 4 postinfection and homogenized them by using FastPrep Homogenizer (MP Biomedicals, https://www.mpbio.com) in PBS. After centrifugation of the supernatants at 5,000 × *g* for 10 min at 4°C, we analyzed them by using plaque assay on Vero cells. We collected whole blood from animals anesthetized with ketamine/xylazine by using heart puncture before cervical dislocation. We prepared serum samples by using incubation at 37°C for 10 min and centrifugation at 5,000 × *g* for 10 min. We used serum samples directly to determine alanine transaminases by using an alanine color endpoint assay (MaxDiscovery; Bio Scientific, http://www.biooscientific.com), or we stored the samples at −20°C. We generated postinfectious serum directed against BRBV by challenging C57BL/6 mice with 10^5^ PFU/animal. Fourteen days after infection, we harvested the serum. Because of the lethality of DHOV, we used Mx1^+/+^ mice for the infection and production of specific antiserum directed against DHOV, as described previously (*18*).

We performed antibody treatment of the animals by intraperitoneal injection. To deplete IFN-γ, we injected 0.5 mg of IFN-γ monoclonal antibody (mAb) (XMG1.2; Biolegend, https://www.biolegend.com) at 1 day preinfection and 2 days postinfection. We achieved blockage of the type I IFN receptor (IFNAR) by treating the mice with 1 mg of anti-IFNAR-1 mAb (MAR1–5A3; BioXCell, https://bxcell.com) at 1 day preinfection and 1 day postinfection. To deplete natural killer (NK) cells, we treated IFNAR^−/−^ mice infected with 100 PFU of BRBV with 0.25 mg of NK1.1 mAb (PK136, BioXcell) at 3 days preinfection and 1 day postinfection. At 4 days postinfection, we harvested organs and used fluorescence-activated cell sorting analysis to determine virus titers and confirm the depletion of NK1.1^+^ cells.

We administered 20 mg or 40 mg of ribavirin (5 mg/mL in 0.9% NaCl; Sigma-Aldrich, https://www.sigmaaldrich.com) per kilogram bodyweight each day intraperitoneally, starting immediately postinfection. We mock-treated control animals with 0.9% NaCl only.

For histologic analysis, we harvested the organs, which we washed once in PBS and then fixed in 4% formaldehyde in PBS overnight. For cryoprotection, we incubated organs once in 15% sucrose (in H_2_O) at 4°C for 4 h and afterward in 30% sucrose at 4°C overnight. After embedding in OCT medium (Tissue-Tek; Sakura, https://www.sakuraus.com), we performed 5 µm cryosections and stained them with hematoxylin and eosin.

### Western Blot Analysis and Antibodies

We infected Vero cells with the indicated viruses (MOI 0.25) for 24 h and then lysed them in Passive Lysis Buffer (Promega, https://www.promega.com). We denaturated proteins in Lämmli buffer and incubated them at 95°C for 5 min. We separated the protein lysates by using 12% SDS-polyacrylamid gel electrophoresis and transferred them onto a PVDF membrane (Millipore Sigma, http://www.emdmillipore.com). We detected viral proteins by using polyclonal mouse antisera. We used β-actin–specific rabbit antiserum (Sigma-Aldrich) as an internal control. We detected primary antibodies by using fluorescent-labeled anti-mouse secondary antibodies (LI-COR, https://www.licor.com).

To test the antiserum for virus neutralization, we prepared serial dilutions of the polyclonal mouse serum in PBS and incubated them with a fixed amount of 100 PFU of BRBV for 1 h at room temperature. To establish a control, we incubated virus with PBS or an unspecific mouse serum. We transferred the virus–serum mixture onto Vero cells and performed a plaque assay. We normalized the PFU of the antibody-treated viruses to the control virus.

### Real-Time Reverse Transcription PCR 

RNA was extracted (NucleoSpin RNA kit; Macherey-Nagel, https://www.mn-net.com) from infected cells and subjected to cDNA synthesis (QuantiTect Reverse Transcription Kit; QIAGEN, https://www.qiagen.com). We performed real-time reverse transcription PCR (RT-PCR) by using 10 ng cDNA in a SYBR Green assay (QuantiTect PCR Kit, QIAGEN) with primers specific for human IFN-β (Hs_IFNB1_1, QIAGEN) and human γ-actin (Hs_ACTG1_1, QIAGEN). We normalized cycle threshold values to actin (ΔCT) and plotted them relative to the ΔCT values of the mock-treated control (2^–ΔΔCT^). We detected viral transcripts of BRBV and DHOV by using panspecific Thogotovirus primers (FW: TTCAATGAATGYTTGGACCCAGATGC [segment 2, nucleotides 940–965]; RW: TTGWACATYCCCATGAACAT [segment 2, nucleotides 1,210–1,229]) in a conventional RT-PCR; we detected the products by using an ethidium bromide–stained agarose gel.

### Statistical Analyses

We analyzed all data with Prism 7 software (GraphPad, https://www.graphpad.com). We performed statistical analysis of viral titers on log-transformed values by using a 1-way analysis of variance with a Tukey multiple comparison test (for >3 groups) or a 2-sided *t*-test (for 2 groups). We plotted viral titers either on a log scale (geometric mean) or log transformed on a linear scale (mean + SD). For weight loss, we calculated SEM.

## Results

### BRBV Sensitivity to Type I and Type II IFN

**Figure 1:**
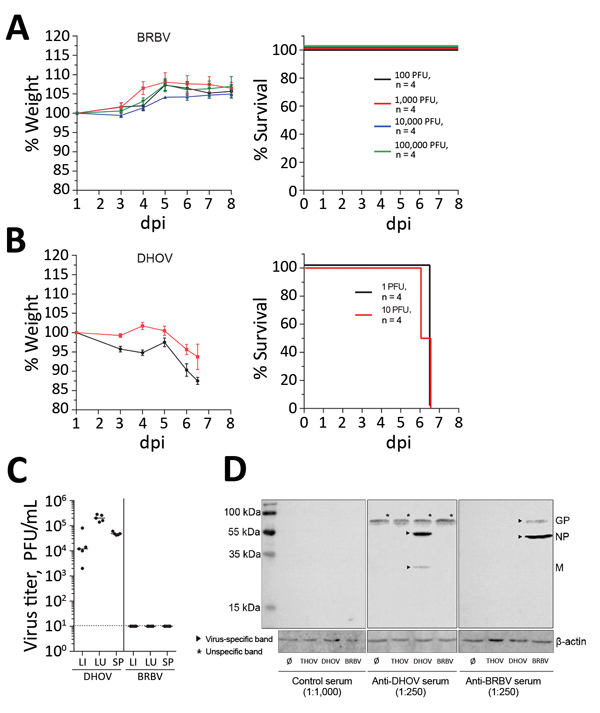
Results of virulence testing of BRBV in immunocompetent mice. A, B) B6 mice (n = 4) infected intraperitoneally with the indicated doses of BRBV (A) or DHOV (B) were monitored for weight (mean +SEM) and survival. Animals were euthanized if they lost >25% body weight or showed severe illness. C) For virus growth, B6 mice (n = 5) were infected intraperitoneally with 100 PFU of DHOV and 1,000 PFU of BRBV. After 4 days, liver, lung, and spleen were harvested and a plaque assay performed. Geometric means are shown; dotted line indicates detection limit. D) Serum samples from BRBV- or DHOV-infected mice were pooled at 14 dpi. Vero cells were mock-treated or infected (multiplicity of infection 0.25) with BRBV, DHOV, and THOV and Western blot analysis of lysates from infected cells performed with antiserum at the indicated dilutions. Molecular weight and identity of the viral antigens are indicated. BRBV, Bourbon virus; DHOV, Dhori virus; dpi, days postinfection; GP, glycoprotein; LI, liver; LU, lung; M, matrix protein; NP, nucleoprotein; SP, spleen; THOV, Thogotovirus; ∅, mock-treated (control).

We studied the virulence of BRBV by infection of C57BL/6 (B6) mice with high challenge doses of BRBV that did not result in disease (Figure 1, panel A), as reported previously (*8*). The lack of pathogenicity is surprising because related Thogotoviruses regularly cause generalized severe infections in IFN-competent laboratory mice (*11*). Infection of B6 mice with the closely related DHOV led to severe illness and early death (Figure 1, panel B). Virus replication was detectable in liver, lung, and spleen of DHOV-infected but not BRBV-infected animals (Figure 1, panel C). Successful BRBV infection was confirmed by seroconversion (Figure 1, panel D) and generation of neutralizing antibodies (Appendix Figure).

**Figure 2:**
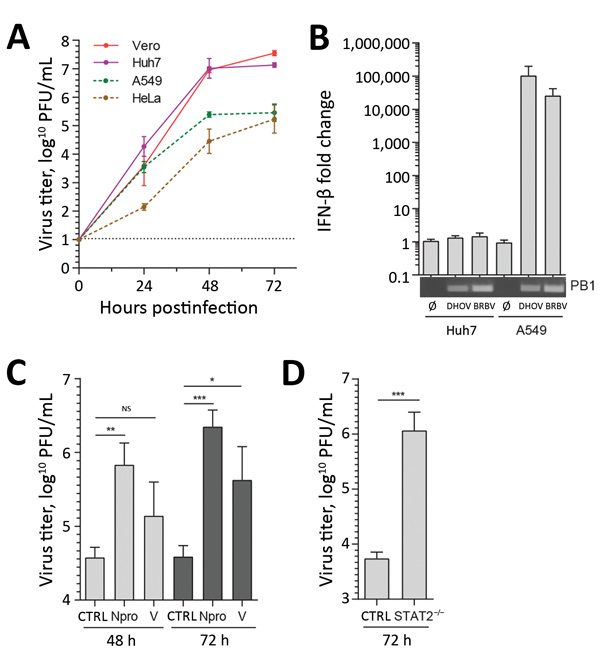
Results of sensitivity testing of BRBV to type I IFN–induced antiviral state in cell culture. A) Vero, Huh7, A549, and HeLa cells was infected with BRBV (multiplicity of infection [MOI] 0.001). At the indicated time points, the supernatants were harvested and viral titers determined. B) Huh7 or A549 were infected (MOI 0.25) with BRBV or DHOV for 16 h. Whole RNA was extracted and IFN-β and actin transcripts detected by real-time reverse transcription PCR. Changes in IFN-β transcripts were calculated in comparison to mock-treated cells. A conventional reverse transcription PCR assay with panspecific primers for viral segment 2 (PB1) was performed to control the infection. C, D) Defects in IFN induction or signaling enhancing BRBV propagation. Parental A549 and A549 cells stably expressing Npro of bovine viral diarrhea virus or the V protein of simian virus 5 (C), as well as (D) human skin fibroblast control cultures or with a defect in STAT2 infected with BRBV (MOI 0.001). Culture supernatants were collected and viral titers determined. Shown are the arithmetic means (+SD) of log-transformed values of 3 independent experiments. Statistical analyses were performed with a 1-way analysis of variance (Tukey multiple comparison test) (C) or a 2-tailed *t*-test (D). BRBV, Bourbon virus; CTRL, control; DHOV, Dhori virus; IFN, interferon; NS, nonsignificant; ∅, mock-treated (control). ***p<0.001; **p<0.01; *p<0.05.

BRBV replicated to high titers in cells that are defective in IFN responses such as simian Vero or human Huh7 cells (*13*,*19*), as previously reported (*8*) (Figure 2, panel A). In IFN-responsive human A549 and HeLa cells, virus growth was reduced by 2 logs (Figure 2, panel A). Real-time RT-PCR analysis showed that BRBV and DHOV strongly induced IFN-β expression in A549 cells, whereas Huh7 cells were unresponsive, as reported previously (*13*). Both cell lines were efficiently infected, as demonstrated by viral PB1 gene expression (Figure 2, panel B). To confirm the role of type I IFN in the suppression of BRBV, we infected A549 cells that were stably overexpressing the IFN-antagonistic Npro protein of bovine viral diarrhea virus or the V protein of simian virus 5. Npro inhibits IFN synthesis by targeting transcription factor IRF3 (*20*), and the V protein blocks IFN signaling by targeting STAT1 for degradation (*21*). Both cultures showed enhanced BRBV replication compared with the parental A549 control cells (Figure 2, panel C), indicating that BRBV-induced IFN elicited an antiviral state that suppressed virus propagation. Enhanced growth of BRBV was also observed in transformed dermal fibroblast cultures obtained from an IFN-nonresponsive person with a genetic defect in STAT2 (*14*) (Figure 2, panel D). BRBV clearly is highly sensitive to the antiviral state induced by IFN.

**Figure 3:**
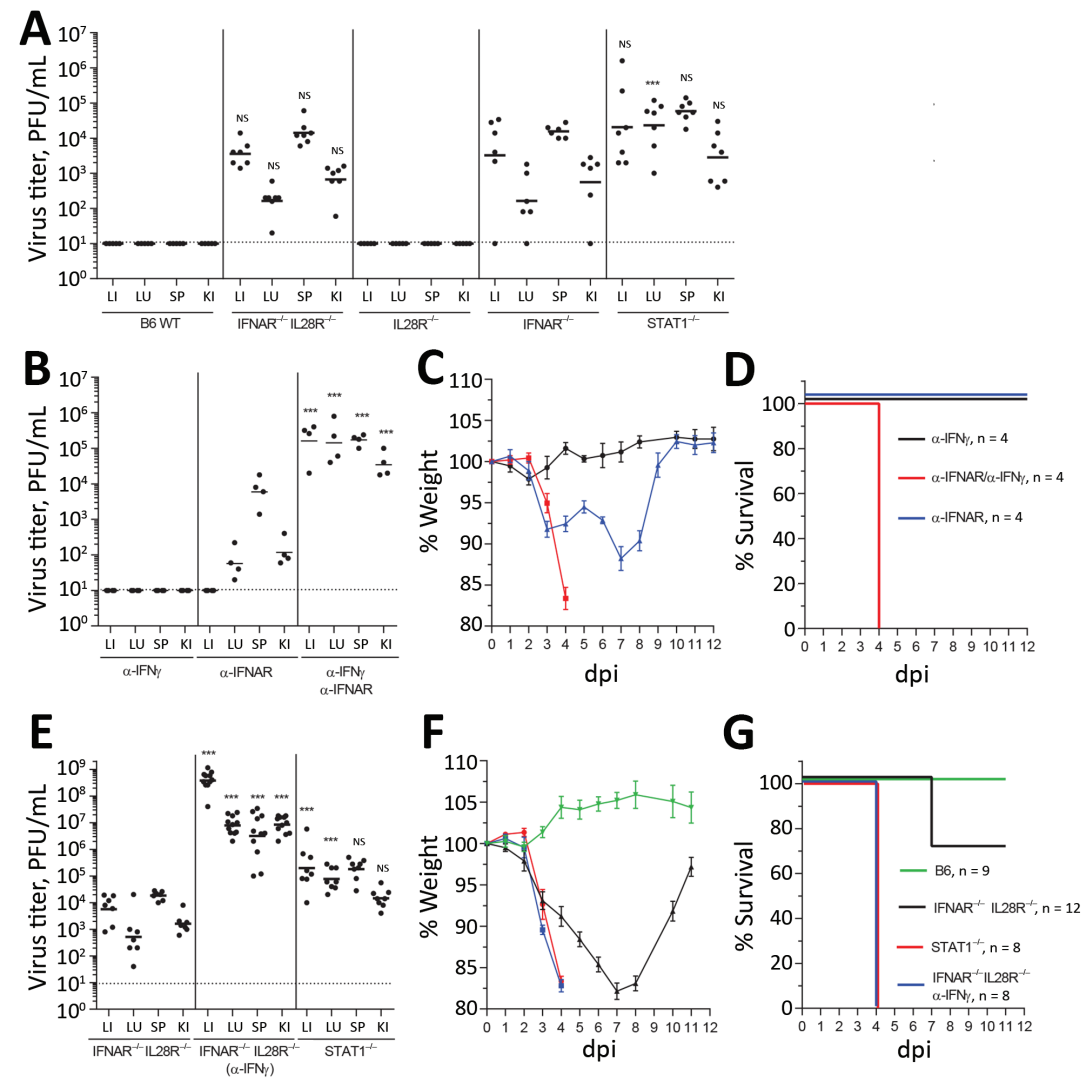
Immune response to BRBV in vivo. A) B6 WT (n = 5) mice or animals with a knockout in IFNAR (IFNAR^−/−^) (n = 6), IL28R^−/−^ (n = 6), IFNAR^−/−^ IL28R^−/−^ double knockout (n = 6), or STAT1^−/−^ (n = 7) infected intraperitoneally with BRBV (1000 pfu). Liver, lung, spleen, and kidney were harvested at day 4 and viral titers determined. B) B6 WT animals (n = 4/group) treated with monoclonal antibodies directed against IFNAR-1 (0.5 mg/mouse 24 h before and 24 h after infection) or against IFN-γ (1 mg/mouse 24 h before and 48 h after infection) and infected with BRBV (100 PFU) for 4 d. C, D) B6 WT animals (4 per group) treated as in panel B and weight loss (mean +SEM) and survival monitored. Animals were euthanized if they lost >25% bodyweight or showed severe clinical signs. E) IFNAR^−/−^ IL28R^−/−^ mice treated with α-IFNγ antibody (n = 11) or left untreated (n = 7) as described in panels B–D and STAT1^−/−^ (n = 8) animals infected with BRBV (100 PFU). At day 4, postinfection viral titers were determined. F, G) The mice (E) together with additional B6 WT (n = 9) were monitored for weight loss (mean +SEM) and survival as in panels C and D. In panels A, B, and E, geometric means are displayed and dotted lines indicate detection limits. Statistical analyses were performed on log-transformed values with a 1-way analysis of variance (Tukey multiple comparison test). Statistics are presented in comparison to the respective organs of IFNAR^−/−^ (A), α-IFNAR (B), or IFNAR^−/−^ IL28R^−/−^ (E). BRBV, Bourbon virus; dpi, days postinfection; IFN, interferon; IFNAR, type I interferon receptor; KI, kidney; LI, liver; LU, lung; NS, nonsignificant; SP, spleen; WT, wild-type. ***p<0.001.

We obtained similar results in vivo using IFN-nonresponsive animals. Mice lacking IFN-α/β receptor expression (IFNAR^−/−^) cannot respond to type I IFN, whereas mice devoid of both IFN-α/β and IFN-λ receptor expression (IFNAR^−/−^ IL28R^−/−^ double knockout mice) are nonresponsive to type I and type III IFNs (*17*). Although both types of mice supported BRBV growth in liver, lung, spleen, and kidney (Figure 3, panel A), virus growth was not detectable in single IL28R^−/−^ mice, indicating that type III IFN plays a minor role. Unexpectedly, STAT1^−/−^ mice were even more susceptible than the IFN receptor–deficient animals, and BRBV grew to higher titers (Figure 3, panel A). We reasoned that IFN-γ (type II IFN) might have a role in controlling virus replication because, like IFN-α/β, IFN-γ signaling relies on STAT1 for signal transduction and antiviral activity. We therefore treated B6 mice with monoclonal antibodies directed either against IFN-γ or against IFNAR. Blocking IFN-γ had no effect, whereas blocking IFNAR led to detectable BRBV replication in most organs tested except the liver (Figure 3, panel B).Treatment with a mixture of both antibodies massively increased virus growth in all organs, including the liver (Figure 3, panel B), indicating that type I and type II IFNs worked synergistically. The synergistic effect was also reflected in the severity of disease. 

Treatment with IFNAR but not IFN-γ antibodies led to a transient drop in body weight with rapid recovery (Figure 3, panel C). In contrast, bodyweight loss in mice treated with both antibodies was dramatic (Figure 3, panel C), and the animals had to be euthanized 4 days after infection (Figure 3, panel D). The same effect was observed in IFNAR^−/−^ IFNLR^−/−^ double knockout mice treated with IFN-γ antibody (Figure 3, panels E–G). IFN-α/β clearly was the dominant antiviral factor controlling BRBV, but its antiviral effect was potentiated by IFN-γ, which is known to act on myeloid cells (*22*), the main target cells of Thogotoviruses (*23*). NK cells are known as the major producers of IFN-γ early during acute infections (*24*). However, depletion of NK cells by treatment of IFNAR^−/−^ mice with NK1.1 antibody could not elevate virus replication (data not shown), leaving the identity of the IFN-γ–producing cell compartment unclear.

**Figure 4:**
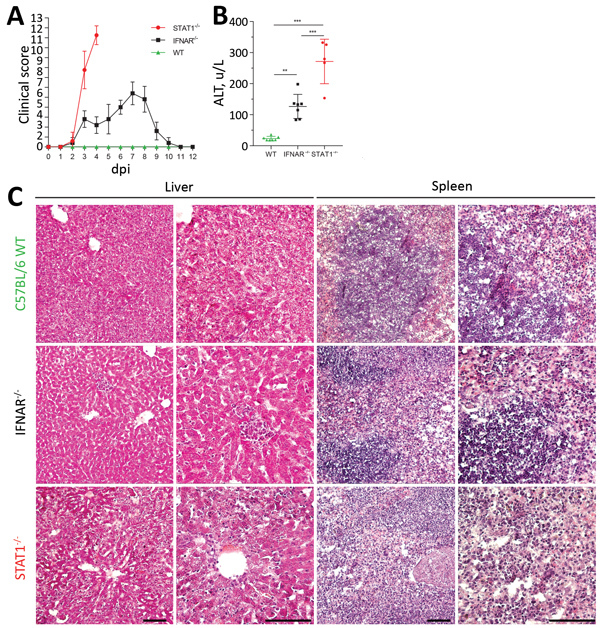
Pathology of BRBV-infected mice. A) B6 WT, IFNAR^−/−^, and STAT1^−/−^ animals (n = 5 for each group) infected intraperitoneally with 100 PFU of BRBV and monitored daily for weight and clinical signs (mean +SD); scoring system described in Appendix Table. B) Serum samples of infected B6 WT (n = 7), IFNAR^−/−^ (n = 7), and STAT1^−/−^ (n = 5) animals harvested at day 4 and analyzed for ALT (mean +SD). Statistical analysis was performed with a 1-way analysis of variance (Tukey multiple comparison test). C) Histologic results showing inflammatory organ damage at 4 dpi. Organs were fixed in 4% formaldehyde and embedded in OCT medium (Tissue-Tek; Sakura, https://www.sakuraus.com); cryosections were then stained with hematoxylin and eosin. Scale bars indicate 100 µm. ALT, alanine aminotransferase; BRBV, Bourbon virus; dpi, days postinfection; IFNAR, type I interferon receptor; WT, wild-type. ***p<0.001; **p<0.01.

We investigated the clinical and pathologic changes in type I (IFNAR^−/−^) and type I and II (STAT1^−/−^) IFN–nonresponsive mice after BRBV infection (Figure 4). Wild-type mice remained asymptomatic throughout the course of infection and showed no gross abnormalities. STAT1^−/−^ mice showed the most severe clinical score and became moribund, whereas IFNAR^−/−^ mice were less severely affected and survived (Figure 4, panel A). Serum alanine aminotransferase levels were elevated as a sign of liver damage (Figure 4, panel B). Histologic liver sections showed massive hepatocellular destructions, including pathologic lesions and infiltrating inflammatory cells (Figure 4, panel C), consistent with acute degenerative hepatitis. Furthermore, the spleens of STAT1^−/−^ animals showed massive necrosis and destruction of the normal architecture (Figure 4, panel C). We also detected infiltrations of granulocytes in lungs and kidneys of the infected STAT1^−/−^ animals, indicating mild inflammation without apparent pathologic changes (data not shown). 

The pathologic and clinical signs observed in mice were in some aspects compatible with the clinical manifestations described for the severe human BRBV cases in 2014 and 2017 (*1*,*3*). The mice showed a high degree of liver damage and elevated liver enzymes, as did the human patients. At later stages, the human patients had acute respiratory complications. Accordingly, the infected STAT1^−/−^ mice showed high virus replication and infiltration of lymphocytes in their lungs accompanied by labored breathing. However, the prominent maculopapular rash that was described in both human patients was not observed in the STAT1^−/−^ mice.

### Antiviral Treatment Effect of Blocking BRBV Replication and Diminishing Pathology

**Figure 5:**
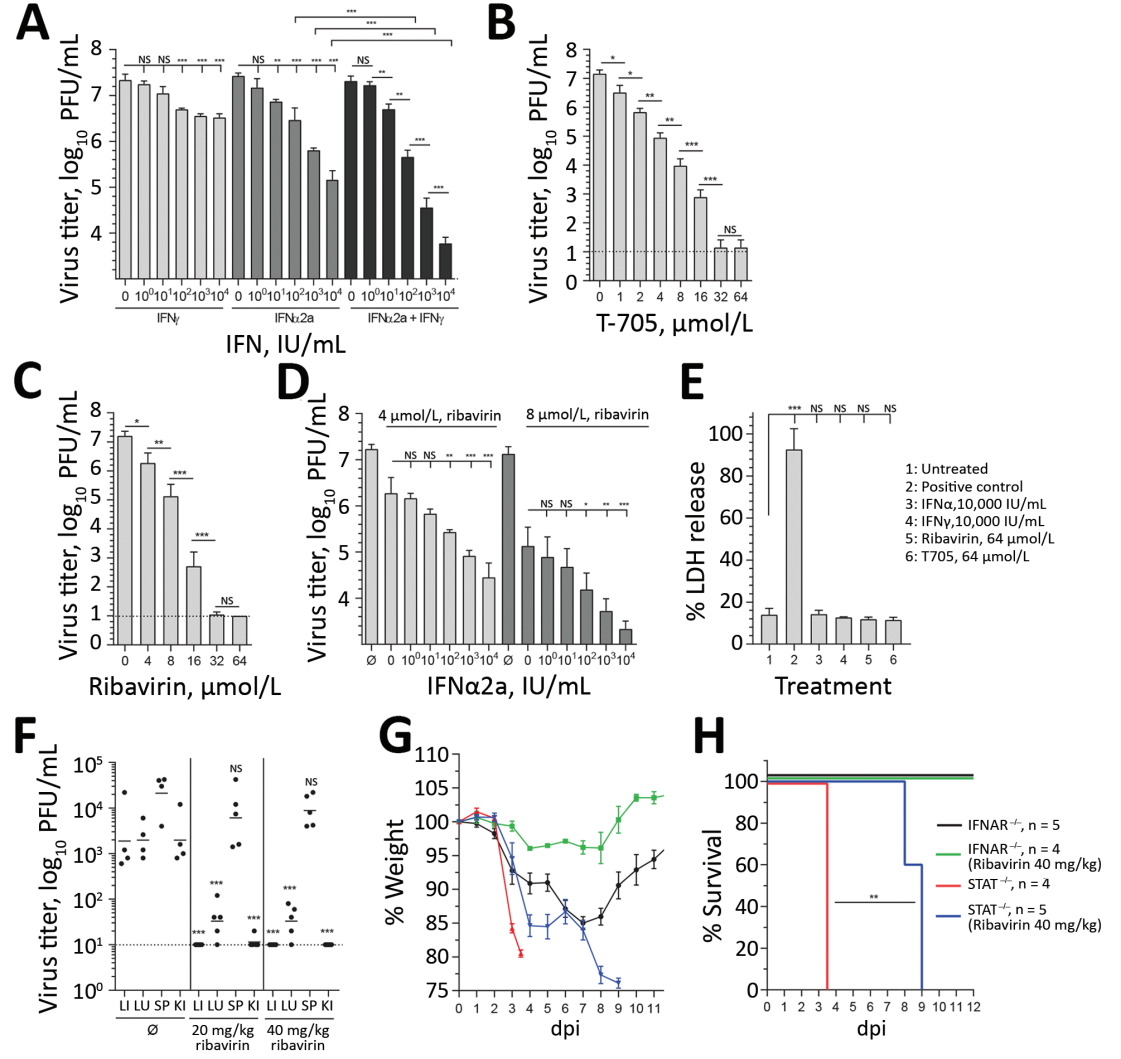
Antiviral treatment against BRBV. A–D) Huh7 cells infected with BRBV (multiplicity of infection 0.001) had viral titers determined at 48 hpi. Shown are the arithmetic means (+SD) of log-transformed values of 3 independent experiments. The cells were treated with increasing amounts of IFN-α2a, IFN-γ, or equal amounts of both IFNs 16 h prior and 2 hpi (A); increasing amounts of the antiviral drugs favipiravir (T705) and ribavirin 2 hpi (B, C); or a combination of ribavirin and IFN-α2a (D). E) To evaluate the cytotoxicity of these compounds, cells were treated with the indicated concentrations for 48 h, or as a positive control the cells were treated with lysis buffer. LDH activity in the supernatant was determined (normalized to positive control [n = 3, mean +SD]). Statistical analyses were performed with a 1-way analysis of variance (Tukey multiple comparison test). F) IFNAR^−/−^ animals (n = 5) treated by intraperitoneal injection with 0.9% NaCl (mock-treated) or 20 or 40 mg/kg/d ribavirin starting 4 hpi with 1,000 PFU of BRBV. At 4 dpi, viral titers were determined in liver, lung, spleen, and kidney. G, H) IFNAR^−/−^ or STAT1^−/−^ mice treated until day 7 dpi with ribavirin (40 mg/kg/d) as in panel F. Weight (mean +SEM) and survival were monitored daily. The animals were euthanized if they lost >25% bodyweight or showed signs of severe illness. H) Statistical analysis for the survival curve performed with a log-rank (Mantel-Cox) test. BRBV, Bourbon virus; dpi, days postinfection; IFN, interferon; IFNAR, type I interferon receptor; hpi, hours postinfection; KI, kidney; LI, liver; LU, lung; LDH, lactate dehydrogenase; NS, nonsignificant; SP, spleen; ∅, mock-treated (control). ***p<0.001; **p<0.01; *p<0.05.

As stated recently by the Centers for Disease Control and Prevention, no antiviral treatment of BRBV disease is available (*25*). Therefore, we tested the protective effect of IFN-α and IFN-γ in Huh7 cells. Treatment with IFN-α led to a 100-fold reduction in viral titers, whereas treatment with IFN-γ was less efficient. Combination of both cytokines led to >1,000-fold reduced virus titers, consistent with a synergistic mode of action (Figure 5, panel A). Treatment with the guanosine analog (ribavirin) and the guanine analog (favipiravir [T705]), both known inhibitors of viral RNA–dependent RNA polymerases (*26*,*27*), resulted in an up to 1 million–fold titer reduction (Figure 5, panels B, C). To evaluate the effect of ribavirin in combination with increasing amounts of IFN-α, we used moderate concentrations that correspond to pharmacologically relevant serum concentrations of 4–8 μmol/L for ribavirin and 100 IU/mL for IFNα (*28*) and observed an up to 1,000-fold reduction of virus replication (Figure 5, panel D). We did not observe cytotoxicity of IFN-α, IFN-γ, ribavirin, or favipiravir (T705) (Figure 5, panel E).

Our mouse models might be suitable to test new treatment regimens to curtail BRBV infections. IFNAR^−/−^ and STAT1^−/−^ mice were infected with a high dose of BRBV and treated daily with ribavirin. The amount of ribavirin used in these experiments was similar to that used in published experiments on influenza virus infections in mice (*26*) and the range of ribavirin treatment of hepatitis C patients (*28*). In our study, treatment reduced BRBV replication in most organs and resulted in efficient titer reductions in liver, lung, and kidney (Figure 5, panel F). Treatment also resulted in reduced weight loss (Figure 5, panel G) and a significant delay of 5 days in the mean time until death in the BRBV-infected STAT1^−/−^ mice (Figure 5, panel H). Our results suggest that ribavirin, possibly in combination with pegylated IFN-α, might be an antiviral treatment option, as in the case of hepatitis C (*29*). 

## Discussion

Characterization of the human BRBV isolate demonstrated a surprisingly strong sensitivity to the type I and type II IFN system. Our results indicate that the few persons to date who had a severe BRBV infection might have had an inborne or transient weakness in their innate antiviral immune response. Also, in case of an acute symptomatic infection with BRBV, our study provides a potential therapeutic option based on the long-approved treatment with ribavirin, possibly in combination with IFN-α. Future analysis of the seroprevalence to BRBV in humans is urgently needed to evaluate the zoonotic spread of the virus in the affected area.

Appendix Additional information about essential role of interferon response in containing human pathogenic Bourbon virus.
