# Supplementary material for: Essential Role of Interferon Response in Containing Human Pathogenic Bourbon Virus
Source: Emerg Infect Dis. 2019 Jul;25(7):1304–13. doi: 10.3201/eid2507.181062 (PMC6590733; doi:10.3201/eid2507.181062)
Supplement: Appendix — Additional information about essential role of interferon response in containing human pathogenic Bourbon virus. [file 18-1062-Techapp-s1.pdf]

Article DOI: https://doi.org/10.3201/eid2507.181062

Essential Role of Interferon Response in Containing Human Pathogenic Bourbon Virus

Appendix

Appendix Table. Clinical scoring parameters

Category	Description	Score
Posture	Normal	0
	Slightly hunched	1
	Hunched	2
Fur	Smooth	0
	Patches of ruffed fur	1
	Completely ruffed fur	2
Eyes	Open	0
	Slightly to moderately closed	1
	Completely closed with secretions	2
Spontaneous behavior	Normal	0
	Reduced activity	1
	No activity	2
Provoked behavior	Immediate response to touch	0
	Moderate response to touch	1
	Little to no response to touch	2
Bodyweight	0%–7.5%	0
	7.5%–15%	1
	>15%	2
Maximum score		12

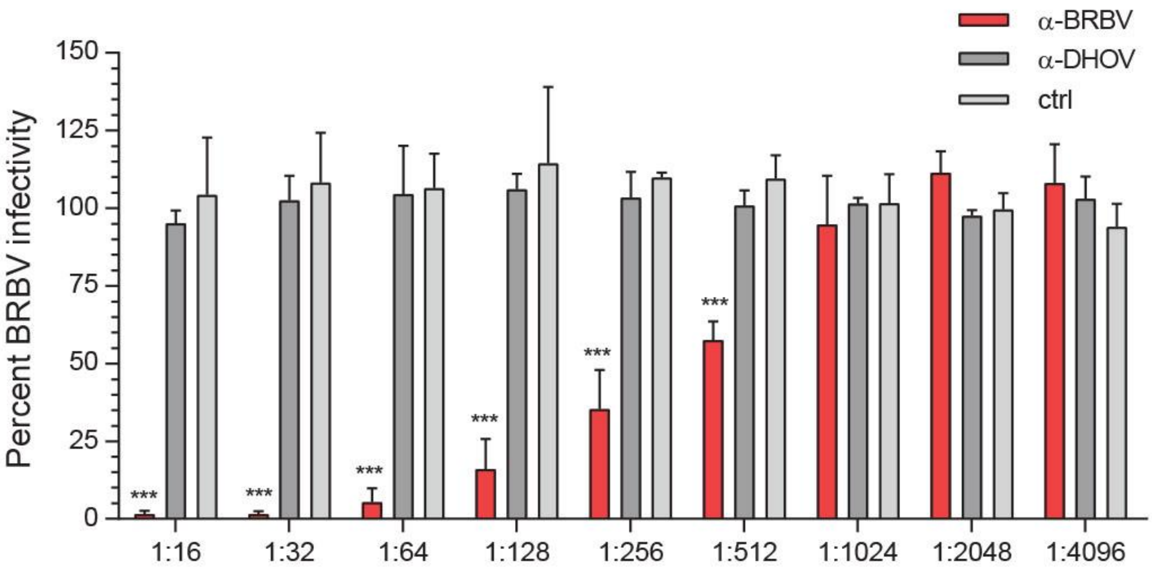

Appendix Figure. BRBV infection of C57BL/6 mice elicit virus-neutralizing antibodies. BRBV (100 PFU) was incubated with the indicated dilutions of sera from mock-infected (CTRL), BRBV- or DHOV-infected mice in a total volume of 100 µL for 1 h at room temperature. Infectivity of the virus–serum mixture was determined by plaque assay. 100% indicates the infectivity of the virus–PBS control mixture. BRBV, Bourbon virus; DHOV, Dhori virus.
